# Comparison of the Decomposition VOC Profile during Winter and Summer in a Moist, Mid-Latitude (Cfb) Climate

**DOI:** 10.1371/journal.pone.0113681

**Published:** 2014-11-20

**Authors:** Shari L. Forbes, Katelynn A. Perrault, Pierre-Hugues Stefanuto, Katie D. Nizio, Jean-François Focant

**Affiliations:** 1 Centre for Forensic Science, University of Technology Sydney, Broadway, NSW, Australia; 2 CART, Organic and Biological Analytical Chemistry Group, Chemistry Department, University of Liège, Liège, Belgium; University of Hawaii at Manoa, United States of America

## Abstract

The investigation of volatile organic compounds (VOCs) associated with decomposition is an emerging field in forensic taphonomy due to their importance in locating human remains using biological detectors such as insects and canines. A consistent decomposition VOC profile has not yet been elucidated due to the intrinsic impact of the environment on the decomposition process in different climatic zones. The study of decomposition VOCs has typically occurred during the warmer months to enable chemical profiling of all decomposition stages. The present study investigated the decomposition VOC profile in air during both warmer and cooler months in a moist, mid-latitude (Cfb) climate as decomposition occurs year-round in this environment. Pig carcasses (*Sus scrofa domesticus* L.) were placed on a soil surface to decompose naturally and their VOC profile was monitored during the winter and summer months. Corresponding control sites were also monitored to determine the natural VOC profile of the surrounding soil and vegetation. VOC samples were collected onto sorbent tubes and analyzed using comprehensive two-dimensional gas chromatography – time-of-flight mass spectrometry (GC×GC-TOFMS). The summer months were characterized by higher temperatures and solar radiation, greater rainfall accumulation, and comparable humidity when compared to the winter months. The rate of decomposition was faster and the number and abundance of VOCs was proportionally higher in summer. However, a similar trend was observed in winter and summer demonstrating a rapid increase in VOC abundance during active decay with a second increase in abundance occurring later in the decomposition process. Sulfur-containing compounds, alcohols and ketones represented the most abundant classes of compounds in both seasons, although almost all 10 compound classes identified contributed to discriminating the stages of decomposition throughout both seasons. The advantages of GC×GC-TOFMS were demonstrated for detecting and identifying trace levels of VOCs, particularly ethers, which are rarely reported as decomposition VOCs.

## Introduction

Studies relating to soft tissue decomposition are typically conducted in mild or warm climates because temperatures in these climates are conducive to recording visible changes in the process of decomposition. It is well known in the field of forensic taphonomy that temperature plays an integral role in the rate of soft tissue decomposition [1]–[7]. This is mostly due to the impact of temperature on biological activity which includes bacteria and fungi [8]–[12], as well as vertebrate [13]–[15] and invertebrate [16]–[20] scavengers.

Although the effect of temperature on soft tissue decomposition has been well-studied, the impact of seasonal variation is less frequently studied, particularly for carcass decomposition as noted by Meyer et al. [21]. The majority of studies to investigate seasonal variation of carcass taphonomy have been carried out with a focus on forensic entomology [19]–[20], [22]–[25]. This is problematic for the field of forensic taphonomy since the decomposition data reported in the literature is predominantly based on degradation in warm climates where biological activity is prevalent. From a forensic perspective, it is also important to study decomposition in cooler or cold climates as human remains can be disposed of during any season of the year. Knowledge of decomposition rates during cooler months is important to ensure accurate estimation of the postmortem interval and subsequent identification of the victim’s remains.

Studies on the seasonal impact on soft tissue decomposition are also important for the success of search and recovery teams that utilize biological detectors such as cadaver-detection or human remains detection (HRD) dogs. While cooler temperatures during winter are known to slow the rate of soft tissue decomposition due to suppression of biological activity (e.g. enzymes, bacteria, and insects), the impact of cooler temperatures on the decomposition odor is mostly based on anecdotal evidence by scent-detection canine handlers. It is typically assumed that on cool days, the odor of decomposition will remain close to the body, making it difficult to detect, whereas on warm days the volatile organic compounds (VOCs), which comprise decomposition odor, will be dispersed in the form of a scent pool [26]. To date, the impact of seasonal variation on the decomposition VOC profile has not been well studied particularly with respect to the composition, number, and abundance of VOCs in decomposition odor in outdoor environments.

Several studies have however investigated decomposition odor in different climatic zones and seasonal temperatures. Vass et al. [27]–[28] developed a Decomposition Odor Analysis Database based on the VOC profile of human remains buried in Tennessee, USA. The study was conducted across all four temperature zones with average temperatures ranging between −7–27°C; however the seasonal variation in VOC profile was not reported. Statheropoulos et al. [29] reported the decomposition VOC profile of two victims recovered near the island of Samos in Greece and stored in body bags in a mortuary in Athens. The victims were recovered from an aquatic environment in December 2002, which predominantly represents the autumn months in the Northern Hemisphere. Although the temperature of the water environment was not reported, the mean ambient temperature during sampling of the decomposition VOC profile in the morgue ranged from 13.75–17.45°C which is consistent with historical average ambient temperatures in Greece for December 2002. Studies have been carried out in Belgium by Brasseur et al. [30] and Dekeirsschieter et al. [31]–[32] and were conducted predominantly during the spring and summer months when temperatures ranged between 3.0–22.2°C. Stadler et al. [33] also investigated the decomposition VOC profile during the spring and summer months in Ontario, Canada where the temperature range was much broader, i.e. 5.15–41.91°C. A recent study conducted by the authors was carried out during the summer months in Sydney, Australia with temperatures ranging from 16.0–31.8°C [34]. This represents the same environment in which the current summer trial was conducted.

Although these studies have reported decomposition VOC profiles over a broad range of temperatures [27]–[37], the impact of seasonal variation has not been investigated within the same outdoor environment even though it has important implications for locating human remains. The purpose of this study was to directly compare the VOC profile of carcass decomposition in a moist, mid-latitude climate across two distinct seasons, i.e. winter and summer. Weather variables other than temperature (specifically rainfall, humidity, solar radiation, and wind speed) were also measured, as these can impact the resultant VOC profile that is produced by carcass decomposition. Based on a laboratory study by Kasper et al. [11], it was hypothesized that the decomposition VOC profile would be reduced with respect to the number and abundance of VOCs produced due to a slowed rate of decomposition in winter when compared to summer. VOCs were analyzed using comprehensive two-dimensional gas chromatography – time-of-flight mass spectrometry (GC×GC-TOFMS) to ensure trace VOCs were detected and identified and a comprehensive VOC profile was determined for each season.

## Materials and Methods

### Experimental study design

The winter and summer trials each utilized four pig (*Sus scrofa domesticus* L.) carcasses weighing approximately 70 kg as analogues for adult human decomposition. Carcasses were purchased from Hawkesbury Valley Meat Processors, a licensed abattoir in Sydney, Australia. Following the guidelines of the Australian Code of Practice for the Care and Use of Animals for Scientific Purposes, animal ethics approval was not required, as the experimental subjects were not killed specifically for the purposes of the research and were purchased postmortem. All pigs were killed using captive-headbolt, the standard procedure employed in Australian abattoirs. Carcasses were transferred to the field site within two hours of death and were placed on the soil surface within minutes of each other. All carcasses were deemed to be in the fresh stage of decomposition at this time.

The field site was private land belonging to the University of Technology Sydney (UTS). The land is zoned for educational and research purposes and the field studies did not involve endangered or protected species; hence no specific permissions were required. The site is located in an open *Eucalyptus* woodland in western Sydney, Australia (33°38S, 150°39E). The soils in this area consist of sandy clay topsoil underlain by shale clays and sandstone bedrock. The topsoil at the study location ranges between pH 4.5–7.0 in winter and pH 5.0–7.5 in summer.

During the winter and summer trials, four carcasses were placed directly on the soil surface and covered with anti-scavenging cages. Four control sites were established for each trial in proximity to the experimental sites to measure the natural VOC profile produced by the surrounding soil and vegetation. The winter trial was carried out between July – October 2013 (a postmortem interval of 106 days), and the summer trial was carried out between January – March 2014 (a postmortem interval of 73 days). The variation in postmortem period represented the degree of time required to study the complete decomposition process from the fresh stage to dry/remains. Weather conditions at the field site were monitored using a Hobo Weather Station equipped with a Hobo U30 No Remote Communication (NRC) data logger (OneTemp, Marleston) and included hourly measurements of ambient temperature (°C), rainfall (mm), relative humidity (%), solar radiation (W/m^2^), wind speed (m/s), gust speed (m/s), and wind direction (ø). The south-eastern region of Australia where the study was conducted is classified by the Köppen Climate Classification System as a moist, mid-latitude (Cfb) climate with warm to hot summers and mild winters [38].

The decomposition stage was characterized by observing and photographing the carrion on each sample collection day and reporting adapted stages originally developed by Payne [39]. Decomposition stages are reported in both experimental days and accumulated degree-days (ADD) to facilitate comparison across the two trials. The sum of the average daily temperatures produced the ADD, which was used to account for differences in the rate of decomposition based on temperature effects [5]. Sample collection was determined based on the rate of decomposition with frequent VOC collection during the first month postmortem and reduced VOC collection as decomposition advanced towards the end stages.

### VOC sample collection

Prior to VOC sample collection, a stainless steel hood was placed over the carrion or control sites and the headspace (gas phase) was allowed to accumulate within the hood for 15 min. A sorbent tube containing Tenax TA and Carbograph 5TD (Markes International Ltd., UK) was connected to the hood via a Swagelok bulkhead connector and the headspace drawn onto the tube at a rate of 100 mL/min for 10 min using an ACTI-VOC low flow sampling pump (Markes International Ltd., UK) (see [34] for diagram of experimental design). The tubes were sealed with brass storage caps, wrapped in foil, and placed in an airtight glass container for transport to the laboratory. Two field blank samples were also collected; one prior to the first sample collection and one at the completion of sample collection on each sampling day, to monitor the natural change in the atmospheric VOC profile at the site. Field blank tubes were transported and stored with sample tubes to account for any contamination and artifacts.

### VOC sample analysis

An internal standard was injected onto each of the sorbent tubes using an automated syringe pipette to ensure injection repeatability. Each injection contained 2 µL of 150 ppm bromobenzene (GC grade, Sigma Aldrich, Australia) in methanol (HPLC grade, Sigma Aldrich, Australia). A Markes Unity 2 Thermal Desorber and Series 2 ULTRA multi-tube autosampler (Markes International Ltd., UK) were used to perform thermal desorption of the sorbent tubes. Connection to the instrument was made via a heated transfer line that was attached to the Pegasus 4D GC×GC-TOFMS (LECO, Australia) via an Ultimate Union Kit (Agilent Technologies, Australia). The first dimension (^1^D) column was a Rxi-624Sil MS (30 m×0.250 mm ID, 1.40 µm film thickness, Restek Corporation, Australia) connected to a second dimension (^2^D) Stabilwax column (2 m×0.250 mm ID, 0.50 µm film thickness, Restek Corporation, Australia). The junction between columns was made using a SilTite µ-Union (SGE Analytical Science, Australia). A constant flow rate of high purity helium carrier gas (BOC, Australia) at 1.00 mL/min was used. The primary oven was held initially at 35°C for 5 min, followed by an increase to 240°C at a rate of 5°C/min, and then held for 5 min (i.e. a total run time of 51 min). The modulator offset was 5°C and the secondary oven temperature offset was 15°C. A 5 s modulation period was used with a 1 s hot pulse time. The MS transfer line was held at 250°C. The mass range was 29–450 amu at an acquisition rate of 100 spectra/s. The ion source temperature was 200°C and the electron ionization energy was −70 eV. A 200 V offset was used above the optimized detector voltage.

### Data processing

Data processing was performed using ChromaTOF (version 4.50.8.0, LECO, Australia). Peak alignment and normalization to the internal standard was performed using the Statistical Compare feature within the ChromaTOF software (LECO, Australia). Alignment was performed on each day based on the two sample classes (control and experimental samples). Automatic baseline smoothing was used with baseline tracking (0.8 offset). A 30 s peak width in ^1^D and 0.15 s peak width in ^2^D was used. A signal-to-noise ratio (S/N) of 150 was used with a minimum similarity match >800 to the NIST (2011) mass spectral library database. Peak identifications were confirmed using retention indices and a range of 84 chemical standards where possible (alkanes, alkylbenzenes, aromatic hydrocarbons, heterocyclic aromatics, chlorinated hydrocarbons, ketones, aldehydes, sulfur-containing compounds, phthalates, primary alcohols, secondary alcohols, fatty acid methyl esters, and Grob test mix compounds). Identified components were required to be present in 50% of the samples within a class to be included in the peak table. The re-searching feature of Statistical Compare also included aligned peaks with a S/N>20 for analytes that were not identified by initial data processing on individual samples using the ChromaTOF peak find function.

Statistical Compare peak tables were further processed in Microsoft Excel by removing column bleed, internal standard artifacts, and field blank compounds. Peak filtering was performed by logarithmic transformation of the normalized peak areas for each compound to which an independent student’s t-test was applied on the experimental and control replicate measurements. Insignificant compounds (p>0.05) were also removed from this list. As it is often found that compounds present in the decomposition VOC profile are also found in control samples (but at lower abundance), this approach allowed for compounds found in common at comparable levels to be excluded. This also allowed for the development of a list of compounds herein referred to as “decomposition VOCs”. Compounds were classified into one or more of the following compound classes: sulfur-containing, nitrogen-containing, aromatic, carboxylic acid, ester, aldehyde, ketone, alcohol, ether, or hydrocarbon. The sum of the relative peak area for each class was averaged across days within a stage and input into The Unscrambler X (version 10.3, CAMO Software, Norway) for analysis by principal component analysis (PCA).

## Results and Discussion

### Weather conditions

The average daily temperature for the winter study demonstrated a general increase as winter transitioned into spring (Figure S1a). The mean temperature throughout the winter study was 14.0°C. The lowest recorded temperature was 1.6°C and the highest recorded temperature was 37.3°C. The winter of 2013 was particularly dry with a total rainfall of only 22 mm on five days of the study period.

The average daily temperature for the summer study was relatively consistent but started to decline with the transition into autumn (Figure S1b). The mean temperature throughout the summer study was 22.1°C. The lowest recorded temperature was 11.4°C and the highest recorded temperature was 42.9°C. Rainfall occurred more frequently during the summer of 2014 with a total accumulation of 120 mm.

Relative humidity (RH) at the site ranged from 8.1–100% in the winter months and 12.7–100% in the summer months. The average relative humidity was comparable between seasons, i.e. 71.1% during winter and 79.2% during summer. Solar radiation measurements ranged from 15.00–130.10 W/m^2^ in winter and 12.38–208.43 W/m^2^ in summer and appeared to have a relationship with the temperatures reported above. Wind speed and gust speed were negligible and varied only slightly above zero.

### Rate of decomposition

The rate of decomposition was visually slower in winter compared to summer. An extended fresh stage was observed during winter on experimental days 0–8 (ADD 71.8) while the fresh stage was only observed on day 0 of the summer trial. An extended bloat stage was also observed in the winter trial on experimental days 9–29 (ADD 82.3–297.0) whereas the bloat stage was only observed on days 1–2 (ADD 28.7–55.5) in the summer trial. Active decay was recorded on days 30–43 (ADD 309.9–463.7) in the winter trial and was extremely rapid in the summer trial, being observed on days 3–6 only (ADD 82.7–161.1). The advanced decay stage was recorded on days 44–79 (ADD 476.0–994.1) in the winter trial and on days 7–23 (ADD 184.4–543.8) in the summer trial. As a result of the cooler temperatures, particularly at night, insect activity was not as prevalent on the pig carcasses in the winter trial and the final stage of decomposition (i.e. dry/remains) was not recorded until day 80 (ADD 1010.8). In comparison, insect activity was very prevalent during the summer months, prompting partial skeletonization and extensive mummification. The final stage of decomposition (i.e. dry/remains) was recorded on experimental day 24 (ADD 564.1) of the summer trial. Although the carcasses in the winter trial took longer to progress through the fresh, bloat, and active decay stages, the ADDs account for the temperature-related variation and each trial was terminated at comparable ADD values (i.e. 1479 for winter and 1613 for summer).

### Comparison of decomposition VOCs and compound classes

The table in Appendix S1 outlines the VOCs detected in both the winter and summer trials. A total of 116 decomposition VOCs were detected during the winter trial and 256 decomposition VOCs were detected during the summer trial. The greater number of VOCs detected during the summer trial is consistent with the faster decomposition process and increased insect activity that resulted from a higher average daily temperature and overall mean ambient temperature. There were no VOCs exclusively identified in the winter trial, as all compounds were detected at some stage in the summer trial. However, the table in Appendix S1 demonstrates that there were many VOCs detected exclusively in the summer trial, which may also be a result of enhanced environmental variables (e.g. increased vegetation and soil microbial activity) during the warmer summer months.

**Figure 1:**
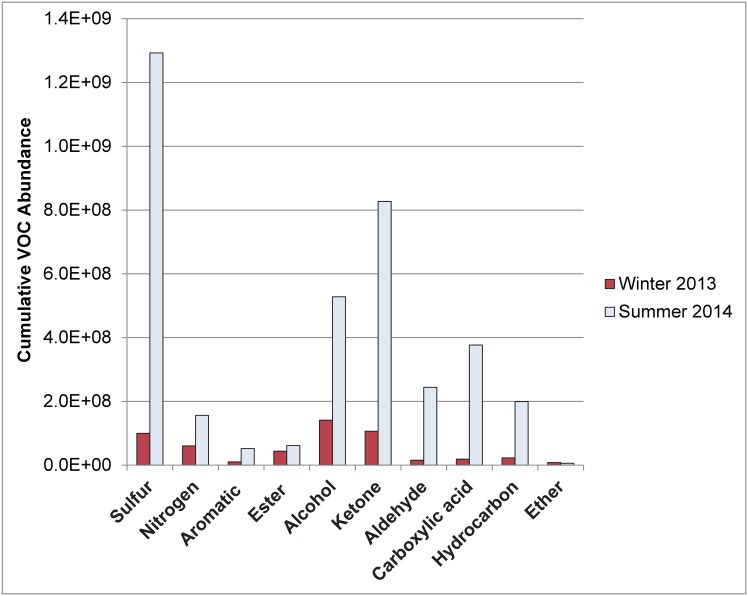
Cumulative VOC abundance by compound class for winter and summer.

The cumulative VOC abundance in each compound class was also typically greater in the summer trial compared to the winter trial (Figure 1) with sulfur-containing compounds representing the highest proportion of VOCs detected. Sulfur-containing compounds were mostly detected during the active and advanced decay stages of the summer trial although they were still prevalent during dry/remains when mummification was recorded. Dimethyl disulfide (DMDS) and dimethyl trisulfide (DMTS) comprised the majority of the sulfur-containing compounds and were detected in all stages of decomposition except the fresh stage, which is consistent with previously reported studies involving both animal and human remains [27]–[34], [36], [40]–[44]. These two sulfur-containing compounds have been identified as important cues for female blowflies with respect to tracing carrion and subsequent oviposition on the remains [45]–[46]. DMDS and DMTS have also been confirmed as being attractive to carrion beetles such as *Nicrophorus vespillo* [40], [47] and *Thanatophilus sinuatus* [48].

Ketones represented another major class of VOCs that were prevalent in the summer trial with a broad range of compounds detected (C_4_–C_14_). Alcohols represented the third most abundant class of VOCs detected in the summer trial. Dominant VOCs in the alcohol class included ethanol, 1-propanol, 2-methyl-1-propanol, 1-butanol, 3-methyl-1-butanol, 2-butanol, 2-pentanol, and phenol. Many of these alcohols have been previously reported in animal and human decomposition odor studies [27], [29], [30]–[34], [41]–[43], [49].

The winter trial also demonstrated a prevalence of sulfur-containing compounds, ketones and alcohols; however, alcohols represented the class with the highest abundance in the overall VOC profile (Figure 1). Alcohols were predominantly detected during the active and advanced decay stages of the winter trial with ethanol and 2-pentanol showing the highest abundances followed by 1-propanol and 2-methyl-1-propanol. The postmortem production of ethanol in tissues with high glucose concentrations is well-known in forensic science, particularly with respect to the issues it causes forensic toxicologists in determining peri-mortem blood alcohol concentrations [50]. Hence, its presence in the VOC profile is not unique. The postmortem production of other short-chain alcohols is also commonly observed in decomposition studies resulting from the bacterial degradation and fermentation of amino acids, fatty acids, and carbohydrates [29], [32]–[33], [42].

Ketones were predominantly detected during the active and advanced stages of decay and, along with hydrocarbons, were one of the few classes of compounds detected during the extended fresh stage in the winter trial. 2-butanone and 2-pentanone were detected during all decomposition stages except dry/remains and demonstrated the highest abundance for the ketones. Previous studies have suggested that ketones and aldehydes are typically detected during the later stages of decomposition [31], [33]; however, compounds from both classes were detected regularly during the bloat, active and advanced decay stages of both the winter and summer trials.

DMDS and DMTS again represented the major sulfur-containing compounds in the winter trial but at reduced abundances compared to the summer trial. Their presence during the bloat stage may account partially for the insect activity that resulted on all carcasses as insect larvae were first observed on day 9 of the winter trial (representing the beginning of the bloat stage). The presence of polysulfides in the decomposition VOC profile is attributed to the chemical degradation of sulfur-containing amino acids in soft tissue and also from the stimulation of enteric and soil bacteria during the decomposition process. Decreased DMDS and DMTS detection in the winter trial may therefore be, in part, attributed to reduced biological activity in the soil beneath the pig carcasses. A concurrent study is also being undertaken by the authors to document the seasonality of the decomposition VOC profile in soil with respect to weather and soil factors (e.g. soil pH and soil moisture).

Aromatics and ethers represented the least prevalent classes of compounds detected in both the winter and summer trials. Aromatics have been reported in previous studies involving both animal and human remains but their prevalence may be a result of the detection of indole, an aromatic heterocyclic VOC containing nitrogen [31], [33], [41], [44], [49]. In this study, indole was classified as a nitrogen-containing compound and represented the most abundant compound in this class. Indole was however only detected during the active and advanced decay stages of the summer trial and was either absent or present below the method detection limit during the winter trial. Ethers have been rarely reported in the literature as decomposition VOCs and their detection in this study was a result of the increased sensitivity, detectability and peak capacity of the GC×GC-TOFMS. The only other study to report a large number of ethers in the decomposition VOC profile also used GC×GC-TOFMS [32].

### Comparison of number and abundance of decomposition VOCs

**Figure 2:**
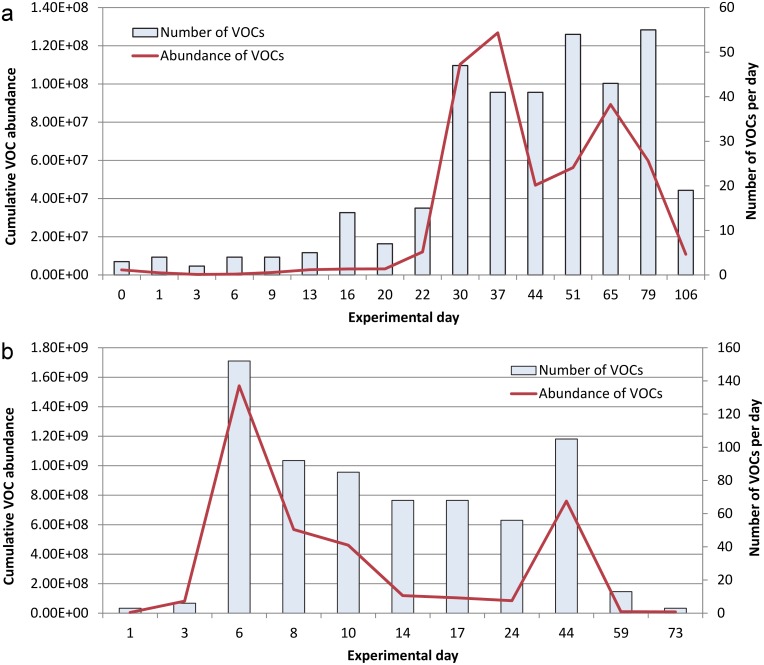
Number and cumulative abundance of VOCs each sampling day for a) winter and b) summer.

The VOC profiles were also different for the winter and summer trials with regard to the number of decomposition VOCs and abundance of those VOCs detected on each sampling day. Figure 2a demonstrates the slow increase in the number and abundance of VOCs with time during the winter trial, consistent with the reduced rate of decomposition resulting from cooler temperatures. There was minimal VOC production during the fresh stage and those VOCs produced were only present at trace levels. Although the number of VOCs increased during the bloat stage, the abundance of those compounds did not increase markedly and were still only detectable at trace levels. Active decay represented the decomposition stage with the highest *abundance* of VOCs as evidenced on days 30 and 37. The abundance of VOCs decreased during advanced decay but increased again on day 65. This may have been a result of a new succession of insects, as although larvae were not visible on the carcasses after active decay, larval masses could be heard on day 65 within the head and internal cavity. Swann et al. [51] reported a similar two-peak cycle for the detection of short-chain volatile fatty acids from carrion decomposition during summer in Western Australia, which they also attributed to larval activity. The highest *number* of VOCs was detected during advanced decay stage on days 51 and 79 demonstrating the dynamic and complex nature of the VOC profile over time. A noticeable reduction in both number and abundance of VOCs was evidenced during the final stage of decomposition (i.e. dry/remains).

VOC production was also minimal during the fresh and bloat stages of the summer trial (Figure 2b). The number and abundance of VOCs increased rapidly with the onset of active decay but declined with the rapid transition to advanced decay. A second increase in VOC abundance was detected on day 44 when the carcasses were characterized as dry/remains with mummification. This second peak may have also resulted from new insect succession due to favorable weather conditions. Typically this stage would not be attractive for fly oviposition but a large accumulation of rainfall between days 33–38 moistened the remaining mummified soft tissue. Observations on day 44 noted that the carcasses were still wet and the odor was more noticeable than the previous sampling day. A large number of empty pupal cases and recently emerged flies were observed on the previous sampling day (i.e. day 24) suggesting that larvae present on the carcasses on day 44 were likely the result of a new succession of insects. Following this peak, the number and abundance of VOCs declined noticeably. The number and abundance of VOCs was proportionally higher in the summer trial when compared to the winter trial.

Appendix S1 and Figure 2 highlight the importance of reporting both the number and abundance of individual VOCs each sampling day to demonstrate the dynamic nature of the decomposition VOC profile. While only semi-quantitative, the abundance of VOCs demonstrates the proportion of VOCs that may be available during each stage of decomposition to biological detectors such as flies, beetles, and cadaver-detection dogs. Although the proportional abundance differed between summer and winter, a similar trend was observed with a rapid increase in VOCs during active decay, followed by a second increase in VOCs later in the decomposition process. It is important to note that the number of VOCs does not always correlate to the abundance and that an increase in the number of VOCs may not alter the overall abundance if all VOCs are present at trace levels for that particular sampling day. This is clearly demonstrated on day 16 of the winter trial (Figure 2a) where a noticeable increase in the number of VOCs did not correlate to an increase in the abundance of VOCs for that sampling day. A comparison of days 37 and 44 of the winter trial (Figure 2a) also demonstrates that the abundance of VOCs can decline even when the number of VOCs remains the same. This finding supports the biochemical degradation of macromolecules into many smaller compounds [2] and the reduced concentration of these compounds that is typically detected as decomposition proceeds. Measuring the abundance of VOCs in air provides a more representative measure of the potential odor available to insects, canines, and other biological detectors.

### Statistical comparison of seasonal data

**Figure 3:**
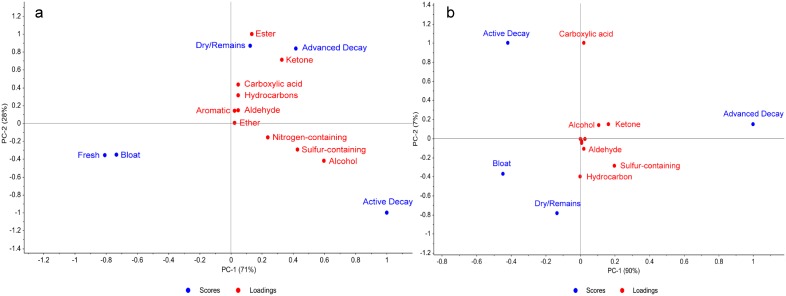
PCA biplot of the calculated scores and loadings for a) winter and b) summer.

Principal component analysis (PCA) was used in order to view the data holistically for each trial based on a biplot of scores (stages of decomposition) and loadings (compound classes). This facilitated assessment of the similarities in seasonal trends because the spatial distribution of the plots could be easily compared. While the data in Figure 1 compared the overall cumulative abundance of each compound class in winter and summer, the PCA data are valuable for identifying the discriminating classes of compounds for each stage of decomposition in both seasons. This is important for compound classes such as nitrogen-containing compounds, esters, carboxylic acids and hydrocarbons which were not considered to be major compound classes in the cumulative data (Figure 1) but were clearly prevalent and discriminatory during certain stages of decomposition (Appendix S1 and Figure 3).

In the winter trial (Figure 3a) a large majority of the compound classes had some effect on the variation between decomposition stages. Due to the majority of the variance being described on PC-1 (71%), the higher presence of alcohols, sulfur-containing compounds, and nitrogen-containing compounds were discriminating for the active decay stage, while esters and ketones also contributed to the discrimination of the advanced decay and dry/remains stages. In the summer trial, (Figure 3b) fewer compound classes discriminated the later stages (carboxylic acids, alcohols, ketone, sulfur-containing compounds and hydrocarbons).

Trends found in common between the two seasons are useful in determining the robustness of the decomposition VOC profile produced year-round. Lower VOC production during the bloat stage is apparent in both biplots (Figures 3a and 3b), which is reflected in Figure 2. Due to the fast onset of the bloat stage in the summer trial, samples were not collected during the fresh stage and do not appear in the PCA analysis. However, in winter it is apparent that the bloat stage clusters with the fresh stage due to the minimal VOC production. For both seasons, ketones and alcohols were discriminatory for the active decay and advanced decay stages. In addition, carboxylic acids were also discriminatory during the advanced decay stage during both winter and summer. Hydrocarbons were a discriminating class in the later stages of decomposition for both trials, particularly in dry/remains.

### Impact of weather conditions on decomposition VOCs

Weather conditions including temperature, rainfall, relative humidity, solar radiation, wind speed, gust speed, and wind direction were measured in this study, as it was believed that they would impact the decomposition VOC profile detected during winter and summer. Since wind speed and gust speed were negligible, these factors were not attributed to the variation in VOC profile observed for both seasons. Relative humidity was also comparable for the winter and summer trials and therefore could not be attributed to the variability of the VOC profile. The high relative humidity could have contributed in part towards the number and abundance of VOCs detected in both trials by maintaining moist and favorable conditions for microbial and insect activity. Kasper et al. [11] reported that humidity (along with temperature) had a significant impact on the decomposition VOC profile of mice (species not reported) carcasses. However, unlike the present study, their study demonstrated a clear distinction in humidity with a warm, humid (80–90% RH) environment compared to a cold, dry (40–60% RH) environment.

Rainfall did appear to impact the VOC profile in the summer months through generation of a new succession of insects on mummified tissue but overall, temperature (and solar radiation, which was likely inter-related to temperature) appeared to have the greatest impact on the rate of decomposition of those variables studied, and subsequent VOC profile produced during each season. It is important to note, however, that temperature may not always be the only major variation between seasons in other climatic zones, particularly where relative humidity, rainfall, and wind speed vary considerably between winter and summer months. Future work should investigate the extent to which each of these variables play a role in the decomposition VOC profile in different seasons. The present study was conducted in a moist, mid-latitude climate with warm to hot summers and mild winters. Other climates under the Köppen Climate Classification System [38] include tropical, dry, polar and highland climates and would likely demonstrate very different impacts on the decomposition process and subsequent VOC profile.

### Benefits of GC×GC-TOFMS

The use of GC×GC-TOFMS has been reported as being advantageous for the analysis of complex matrices such as decomposition odor [30], [32]–[33], [36]–[37]. The benefits of using GC×GC-TOFMS for the chemical profiling of decomposition odor include increased peak capacity, enhanced sensitivity and detectability, and improved characterization of dynamic compound range.

**Figure 4:**
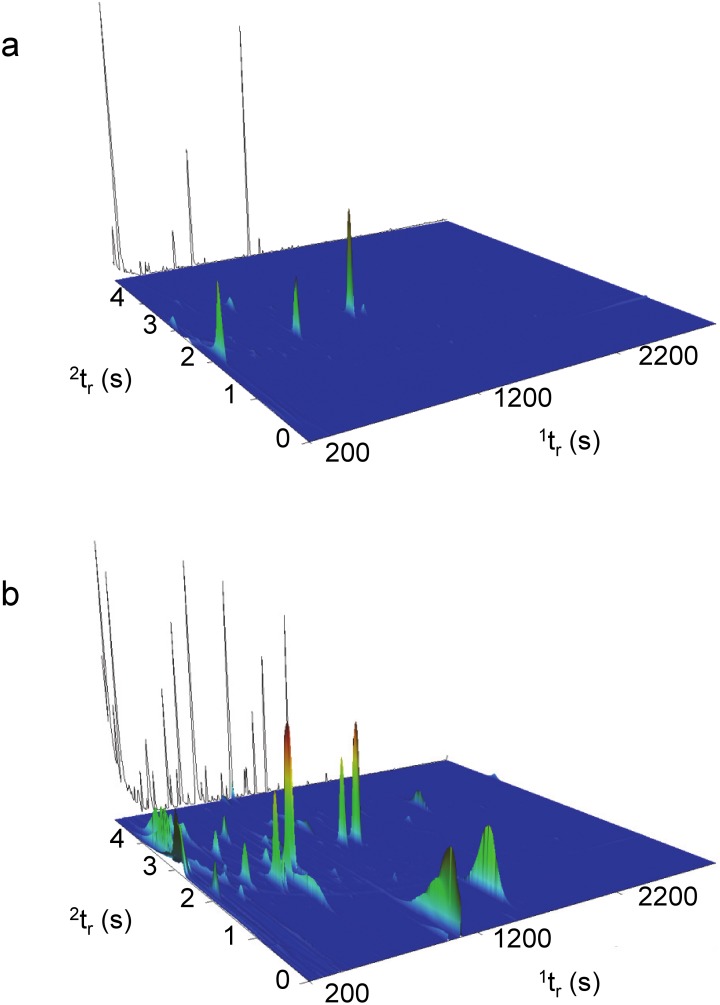
Surface plots (TIC) of active decay stage for a) winter and b) summer including ^1^D trace.

In the present study, GC×GC-TOFMS was valuable for identifying trace VOCs and providing a more comprehensive overview of the decomposition VOC profile. Figure 4 compares the GC×GC surface plot of carcasses in the active decay stage for the winter (day 37) and summer (day 6) trials, (Figures 4a and 4b, respectively). These chromatograms show the first and second dimension retention times (^1^t_r_ and ^2^t_r_, respectively) and are used as representative plots, as they demonstrate the complexity of the decomposition VOC profile when the abundance of VOCs is at a maximum. The solvent peak resulting from the internal standard (bromobenzene in methanol) has been removed for clarity but was included in the original data processing method. This highlights one of several benefits of the accompanying GC×GC software, since it is possible to separate and identify trace or smaller compounds in the second dimension that would be otherwise masked by the large solvent peak in traditional GC-MS.

While the chromatogram for the winter trial (Figure 4a) does not appear particularly complex, 37 individual VOCs were identified as being significant on this experimental day demonstrating the enhanced capability of GC×GC-TOFMS for identifying trace level compounds. A trace at the back of the plot showing the sum of the first dimension (^1^D) demonstrates that many of these trace level compounds would have been lost in baseline noise if traditional GC-MS had been used to analyze these samples. The surface plot for the summer trial (Figure 4b) demonstrates the increased complexity when decomposition proceeds rapidly and a large number of VOCs are generated through the microbial and insect activity associated with the remains. A comparison with the trace at the back of the plot further demonstrates the complexity of the sample and highlights the potential for co-elution of compounds if traditional GC-MS had been used. This is one of the most beneficial aspects of comprehensive two-dimensional GC, as it allows for the separation and identification of peaks that co-elute in the first dimension by separating compounds based on a second property (i.e. polarity) in the second dimension. Moreover, the cryofocus effect, linked to the modulation process, will increase the detection limit of compounds in low concentration, providing a deeper sample screening.

**Figure 5:**
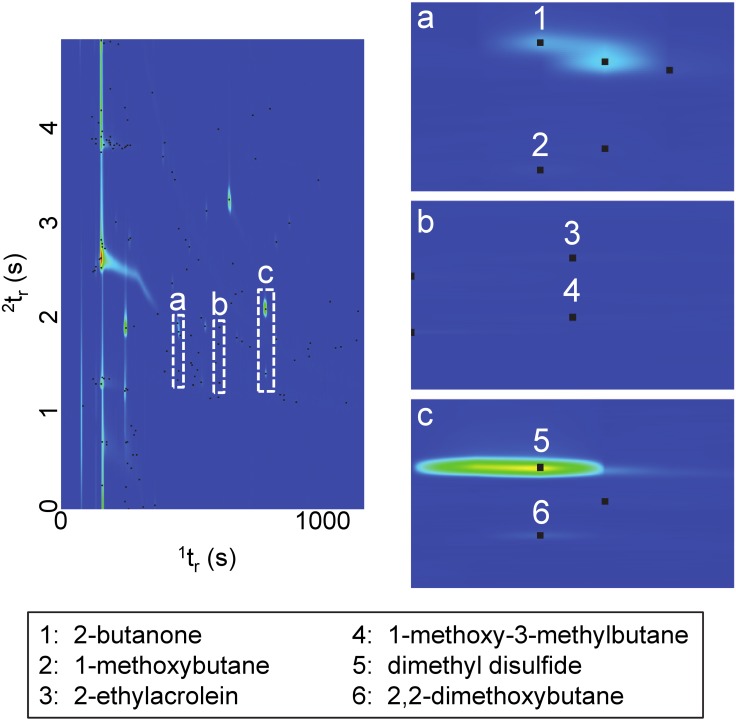
Contour plots (TIC) showing co-elution of a) 1-methoxybutane, b) 1-methoxy-3-methylbutane, and c) 2,2-dimethoxybutane in ^1^D.

The identification of ethers, a rarely reported class of decomposition VOCs, in both seasons resulted from the enhanced sensitivity and increased peak capacity of GC×GC-TOFMS. All of the ethers detected were present at trace levels that may not have been detected using traditional GC-MS. Figure 5a demonstrates the co-elution of 1-methoxybutane (peak 2) with 2-butanone (peak 1). 2-butanone was abundant in all stages of the winter trial except dry/remains and was present during active decay, advanced decay and dry/remains in the summer trial whereas1-methoxybutane was detected at trace levels during the active decay stage of the winter and summer trials. Figure 5b demonstrates the co-elution of 1-methoxy-3-methylbutane (peak 4) with 2-ethylacrolein (peak 3) in the first dimension. During the winter trial, both compounds were detected in the active decay stage demonstrating the importance of the second dimension for clearly separating and identifying these two compounds. 2,2-dimethoxybutane (peak 6 in Figure 5c) was the most prevalent ether and was detected in the bloat, active decay and advanced decay stages for both the winter and summer trials. 2,2-dimethoxybutane co-eluted with DMDS (peak 5 in Figure 5c) in the first dimension explaining why it may not have been previously reported as a decomposition VOC in studies using traditional GC-MS. DMDS was also detected during the bloat, active decay and advanced decay stages of both trials and was typically present in much higher abundances than 2,2-dimethoxybutane.

Only the co-elution of ether compounds (in the first dimension) is reported here, however, other compounds co-eluting in the first dimension were identified in the chromatograms, particularly during the active and advanced decay stages when a large number of VOCs with similar chemical structures were detected. The value of using GC×GC-TOFMS for the analysis of complex matrices is being recognized with an increased number of decomposition VOC studies reporting its use [30], [32]–[33], [36]–[37], [52]. In the present study, the benefits of GC×GC-TOFMS analysis are clearly evidenced by the detection of trace VOCs and the identification of compounds that would have co-eluted or been masked by larger peaks if traditional GC-MS had been employed. This study was therefore able to report a more comprehensive VOC profile, particularly during the winter trial when a reduced number and abundance of decomposition VOCs were produced.

## Conclusion

This study involved a comparison of the decomposition VOC profile generated by pig carcasses placed in a moist, mid-latitude climate during winter and summer months. The winter months of 2013 were particularly dry with a mean ambient temperature approximately 8.0°C lower than that recorded for the summer months of 2014. Humidity was comparable for both seasons but rainfall was greater during the summer months. The variable weather conditions led to a reduced rate of decomposition in winter with extended fresh, bloat and active decay stages compared to summer. As a result, temperature appeared to play the greatest role (of those variables studied) in terms of the decomposition VOC profile produced across both seasons. However, temperature cannot be considered in isolation when studying seasonal variation, since it impacts biological activity that subsequently drives the rate of soft tissue decomposition and the VOC profile produced.

It was originally hypothesized that the decomposition VOC profile would be reduced in winter with respect to the number and abundance of VOCs and this was proven to be correct due to the slower rate of decomposition. However, a similar trend was observed between the winter and summer trials whereby the number and abundance of VOCs rapidly increased with the onset of active decay, followed by a decrease with the onset of advanced decay, and a subsequent increase again during the later stages of decomposition. Overall, sulfur-containing compounds, ketones and alcohols were the most prevalent classes of compounds in both trials, although sulfur-containing compounds clearly dominated the VOC profile during summer. The abundance of sulfur compounds can be related to the increased rate of soft tissue decomposition, increased insect activity, and enhanced soil microbial activity in summer, all of which are known to produce distinctive sulfur compounds such as DMDS and DMTS. While these classes of compounds were prevalent in the overall VOC profile, PCA was valuable for identifying the discriminatory classes for each stage of decomposition. Ketones and alcohols were discriminatory during the active and advanced decay stages whereas hydrocarbons were discriminatory during the dry/remains stage for both winter and summer.

Recent studies have highlighted the importance of using GC×GC-TOFMS for non-target analysis of complex matrices and this was further demonstrated in the present study. The increased peak capacity, sensitivity and detectability, allowed for the detection of trace VOCs as well as identification of ethers that are rarely reported in the decomposition odor profile. Use of this advanced chromatographic technique enabled a more comprehensive VOC profile to be reported demonstrating that even during cooler months, decomposition VOCs can still be present in air at detectable levels.

The findings of this study demonstrate that a considerable number of VOCs were still produced during the winter months of the moist, mid-latitude climate in which the study was conducted. This is likely due to the mild winters typically experienced in this particular climatic zone. The number and abundance of VOCs would suggest that decomposition odor is still prevalent and available to cadaver-detection dogs during cooler months, particularly if the remains are in the active or advanced decay stages. This information can assist canine handlers in determining an optimum search strategy when searching in different seasons. However, these findings need to be interpreted cautiously since it is not yet known which key compounds cadaver-detection dogs respond to and whether these compounds were produced during the winter and summer months of the present study. Future studies will attempt to identify these compounds to further enhance training of cadaver-detection dogs.

## Supporting Information

Figure S1 Average temperature and rainfall data for the study site during the a) winter trial (July – October, 2013) and b) summer trial (January – March, 2014).
(TIF)

Appendix S1 Decomposition VOCs detected during winter (O) and summer (X) for each stage of decomposition.
(DOCX)

## References

[1] Mann RW, Bass WM, Meadows L (1990) Time since death and decomposition of the human body: variables and observations in case and experimental field studies. J Forensic Sci 35: 103–111. 2313251

[2] Gill-King H (1997) Chemical and ultrastructural aspects of decomposition. In: Haglund WD, Sorg MH, editors. Forensic taphonomy: the postmortem fate of human remains. Boca Raton, FL: CRC Press. 93–108.

[3] Clark MA, Worrell MB, Pless JE (1997) Postmortem changes in soft tissue. In: Haglund WD, Sorg MH, editors. Forensic taphonomy: the postmortem fate of human remains. Boca Raton, FL: CRC Press. 151–164.

[4] Bass WM (1997) Outdoor decomposition rates in Tennessee. In: Haglund WD, Sorg MH, editors. Forensic taphonomy: the postmortem fate of human remains. Boca Raton, FL: CRC Press. 181–186.

[5] Megyesi MS, Nawrocki SP, Haskell NH (2005) Using accumulated degree-days to estimate the postmortem interval from decomposed human remains. J Forensic Sci 50: 618–626. 15932096

[6] Vass AA (2011) The elusive universal post-mortem interval formula. Forensic Sci Int 204: 34–40. 20554133 10.1016/j.forsciint.2010.04.052

[7] Zhou C, Byard RW (2011) Factors and processes causing accelerated decomposition in human cadavers – an overview. J Forensic Leg Med 18: 6–9. 21216371 10.1016/j.jflm.2010.10.003

[8] Carter DO, Yellowlees D, Tibbett M (2007) Cadaver decomposition in terrestrial ecosystems. Naturwissenschaften 94: 12–24. 17091303 10.1007/s00114-006-0159-1

[9] Carter DO, Yellowlees D, Tibbett M (2008) Temperature affects microbial decomposition of cadavers (*Rattus rattus*) in contrasting soils. Appl Soil Ecol 40: 129–137.

[10] Hopkins DW (2008) The role of soil organisms in terrestrial decomposition. In: Tibbett M, Carter DO, editors. Soil analysis in forensic taphonomy: chemical and biological effects of buried human remains. Boca Raton, FL: CRC Press. 53–66.

[11] Kasper J, Mumm R, Ruther J (2012) The composition of carcass volatile profiles in relation to storage time and climate conditions. Forensic Sci Int 223: 64–71. 22951222 10.1016/j.forsciint.2012.08.001

[12] Lauber CL, Metcalf JL, Keepers K, Ackermann G, Carter DO, et al (2014) Vertebrate decomposition is accelerated by soil microbes. Appl Environ Microbiol 80: 4920–4929. 24907317 10.1128/AEM.00957-14 PMC4135757

[13] O’Brien RC, Forbes SL, Meyer J, Dadour I (2010) Forensically significant scavenging guilds in the southwest of Western Australia. Forensic Sci Int 198: 85–91. 20171028 10.1016/j.forsciint.2010.01.006

[14] Dabbs GR, Martin DC (2013) Geographic variation in the taphonomic effect of vulture scavenging: the case for Southern Illinois. J Forensic Sci 58: S20–S25. 23181511 10.1111/1556-4029.12025

[15] Young A, Stillman R, Smith MJ, Krostjens AH (2014) An experimental study of vertebrate scavenging behavior in a northwest European woodland context. J Forensic Sci 59: 1333–1342. 24611615 10.1111/1556-4029.12468

[16] Campobasso CP, Di Vella G, Introna F (2001) Factors affecting decomposition and Diptera colonization. Forensic Sci Int 120: 18–27. 11457604 10.1016/s0379-0738(01)00411-x

[17] Archer MS, Elgar MA (2003) Yearly activity patterns in southern Victoria (Australia) of seasonally active carrion insects. Forensic Sci Int 132: 173–176. 12711201 10.1016/s0379-0738(03)00034-3

[18] Joy JE, Liette NL, Harrah HL (2006) Carrion fly (Diptera: Calliphoridae) larval colonization of sunlit and shaded pig carcasses in West Virginia, USA. Forensic Sci Int 164: 183–192. 16497460 10.1016/j.forsciint.2006.01.008

[19] Voss SC, Spafford H, Dadour IR (2009) Annual and seasonal patterns of insect succession on decomposing remains at two locations in Western Australia. Forensic Sci Int 193: 26–36. 19836175 10.1016/j.forsciint.2009.08.014

[20] Sharanowski BJ, Walker EG, Anderson GS (2008) Insect succession and decomposition patterns on shaded and sunlit carrion in Saskatchewan in three different seasons. Forensic Sci Int 179: 219–240. 18662603 10.1016/j.forsciint.2008.05.019

[21] Meyer J, Anderson B, Carter DO (2013) Seasonal variation of carcass decomposition and gravesoil chemistry in a cold (Dfa) climate. J Forensic Sci 58: 1175–1182. 23822087 10.1111/1556-4029.12169

[22] Prado e Castro C, Garcia MD, Martins da Silva P, Faria e Silva I, Serrano A (2013) Coleoptera of forensic interest: a study of seasonal community composition and succession in Lisbon, Portugal. Forensic Sci Int 232: 73–83. 24053868 10.1016/j.forsciint.2013.06.014

[23] Benbow ME, Lewis AJ, Tomberlin JK, Pechal JL (2013) Seasonal necrophagous insect community assembly during vertebrate carrion decomposition. J Med Entomol 50: 440–450. 23540134 10.1603/me12194

[24] Battan Horenstein M, Rosso B, Garcia MD (2012) Seasonal structure and dynamics of sarcosaprophagous fauna on pig carrion in a rural area of Cordoba (Argentina): their importance in forensic science. Forensic Sci Int 217: 146–156. 22138029 10.1016/j.forsciint.2011.10.043

[25] Arnaldos MI, Romera E, Presa JJ, Luna A, Garcia MD (2004) Studies on seasonal arthropod succession on carrion in the southeastern Iberian Peninsula. Int J Legal Med 118: 197–205. 15114485 10.1007/s00414-004-0446-3

[26] Stejskal SM (2013) Death, decomposition and detector dogs: from science to scene. Boca Raton, FL: CRC Press.

[27] Vass AA, Smith RR, Thompson CV, Burnett MN, Wolf DA, et al (2004) Decompositional odor analysis database. J Forensic Sci 49: 760–769. 15317191

[28] Vass AA, Smith RR, Thompson CV, Burnett MN, Dulgerian N, et al (2008) Odor analysis of decomposing buried human remains. J Forensic Sci 53: 384–391. 18366571 10.1111/j.1556-4029.2008.00680.x

[29] Statheropoulos M, Spiliopouiou C, Agapiou A (2005) A study of volatile organic compounds evolved from the decaying human body. Forensic Sci Int 153: 147–155. 16139103 10.1016/j.forsciint.2004.08.015

[30] Brasseur C, Dekeirsschieter J, Schotsmans EM, de Koning S, Wilson AS, et al (2012) Comprehensive two-dimensional gas chromatography-time-of-flight mass spectrometry for the forensic study of cadaveric volatile organic compounds released in soil by buried decaying pig carcasses. J Chromatogr A 1255: 163–170. 22520639 10.1016/j.chroma.2012.03.048

[31] Dekeirsschieter J, Verheggen FJ, Gohy M, Hubrecht F, Bourguignon L, et al (2009) Cadaveric volatile organic compounds released by decaying pig carcasses (Sus domesticus L.) in different biotopes. Forensic Sci Int 189: 46–53. 19423246 10.1016/j.forsciint.2009.03.034

[32] Dekeirsschieter J, Stefanuto PH, Brasseur C, Haubruge E, Focant JF (2012) Enhanced characterization of the smell of death by comprehensive two-dimensional gas chromatography - time-of-flight mass spectrometry (GCxGC-TOFMS). PLoS One 7: e39005. 22723918 10.1371/journal.pone.0039005 PMC3377612

[33] Stadler S, Stefanuto PH, Brokl M, Forbes SL, Focant JF (2013) Characterization of volatile organic compounds from human analogue decomposition using thermal desorption coupled to comprehensive two-dimensional gas chromatography-time-of-flight mass spectrometry. Anal Chem 85: 998–1005. 23215054 10.1021/ac302614y

[34] Forbes SL, Perrault KA (2014) Decomposition odour profiling in the air and soil surrounding carrion vertebrate carrion. PLoS One 9: e95107. 24740412 10.1371/journal.pone.0095107 PMC3989314

[35] Perrault KA, Stuart BH, Forbes SL (2014) A longitudinal study of decomposition odour in soil using sorbent tubes and solid phase microextraction. Chromatogr 1: 120–140.

[36] Stefanuto P-H, Perrault K, Stadler S, Pesesse R, Brokl M, et al (2014) Reading cadaveric decomposition chemistry with a new pair of glasses. ChemPlusChem 79: 786–789.

[37] Focant J-F, Stefanuto P-H, Brasseur C, Dekeirsschieter J, Haubruge E, et al (2013) Forensic cadaveric decomposition profiling by GCxGC-TOFMS analysis of VOCs. Chem. Bull. Kazakh Natl. Univ. 4: 177–186.

[38] Peel MC, Finlayson BL, McMahon TA (2007) Updated world map of the Köppen-Geiger climate classification. Hydrol Earth Syst Sci 11: 1633–1644.

[39] Payne JA (1965) A summer carrion study of the baby pig *Sus scrofa* Linnaeus. Ecology 46: 592–602.

[40] Kalinova B, Podskalska H, Ruzicka J, Hoskovec M (2009) Irresistible bouquet of death–how are burying beetles (Coleoptera: Silphidae: Nicrophorus) attracted by carcasses. Naturwissenschaften 96: 889–899. 19404598 10.1007/s00114-009-0545-6

[41] Hoffman EM, Curran AM, Dulgerian N, Stockham RA, Eckenrode BA (2009) Characterization of the volatile organic compounds present in the headspace of decomposing human remains. Forensic Sci Int 186: 6–13. 19203852 10.1016/j.forsciint.2008.12.022

[42] Statheropoulos M, Agapiou A, Spiliopouiou C, Pallis GC, Sianos E (2007) Environmental aspects of VOCs evolved in the early stages of human decomposition. Sci Total Environ 385: 221–227. 17669473 10.1016/j.scitotenv.2007.07.003

[43] DeGreeff LE, Furton KG (2011) Collection and identification of human remains volatiles by non-contact, dynamic airflow sampling and SPME-GC/MS using various sorbent materials. Anal Bioanal Chem 401: 1295–1307. 21695377 10.1007/s00216-011-5167-0

[44] Cablk ME, Szelagowski EE, Sagebiel JC (2012) Characterization of the volatile organic compounds present in the headspace of decomposing animal remains, and compared with human remains. Forensic Sci Int 220: 118–125. 22424672 10.1016/j.forsciint.2012.02.007

[45] Paczkowski S, Schutz S (2011) Post-mortem volatiles of vertebrate tissue. Appl Microbiol Biotechnol 91: 917–935. 21720824 10.1007/s00253-011-3417-x PMC3145088

[46] LeBlanc HN, Logan JG (2010) Exploiting insect olfaction in forensic entomology. In: Amendt J, Campobasso CP, Goff ML, Grassberger M, editors. Current concepts in forensic entomology. New York: Springer. 205–222.

[47] Podskalská H, Růžička J, Hoskovec M, Sálek M (2009) Use of infochemicals to attract carrion beetles into pitfall traps. Entomol Exp Appl 132: 59–64.

[48] Dekeirsschieter J, Frederickx C, Lognay G, Brostaux Y, Verheggen FJ, et al (2013) Electrophysiological and behavioral responses of Thanatophilus sinuatus Fabricius (Coleoptera: Silphidae) to selected cadaveric volatile organic compounds. J Forensic Sci. 58: 917–23. 10.1111/1556-4029.12123 23822801

[49] Lorenzo N, Wan TL, Harper RJ, Hsu Y-L, Chow M, et al (2003) Laboratory and field experiments used to identify *Canis lupus* var. *familiaris* active odor signature chemicals from drugs, explosives, and humans. Anal Bioanal Chem 376: 1212–1224. 12845400 10.1007/s00216-003-2018-7

[50] Collison IB (2005) Elevated postmortem ethanol concentrations in an insulin-dependent diabetic. J Anal Toxicol 29: 762–764. 16419416 10.1093/jat/29.7.762

[51] Swann L, Forbes SL, Lewis SW (2009) Observations of the temporal variation in chemical content of decomposition fluid: A preliminary study using pigs as a model system. Aust J Forensic Sci 42: 199–210.

[52] Tipple CA, Caldwell PT, Kile BM, Buessman DJ, Rushing B, et al (2014) Comprehensive characterization of commercially available canine training aids. Forensic Sci Int 242: 242–254. 25093917 10.1016/j.forsciint.2014.06.033

