# Supplementary figures and images for: Comparison of the Decomposition VOC Profile during Winter and Summer in a Moist, Mid-Latitude (Cfb) Climate
Source: PLoS One. 2014 Nov 20;9(11):e113681. doi: 10.1371/journal.pone.0113681 (PMC4239107; doi:10.1371/journal.pone.0113681)

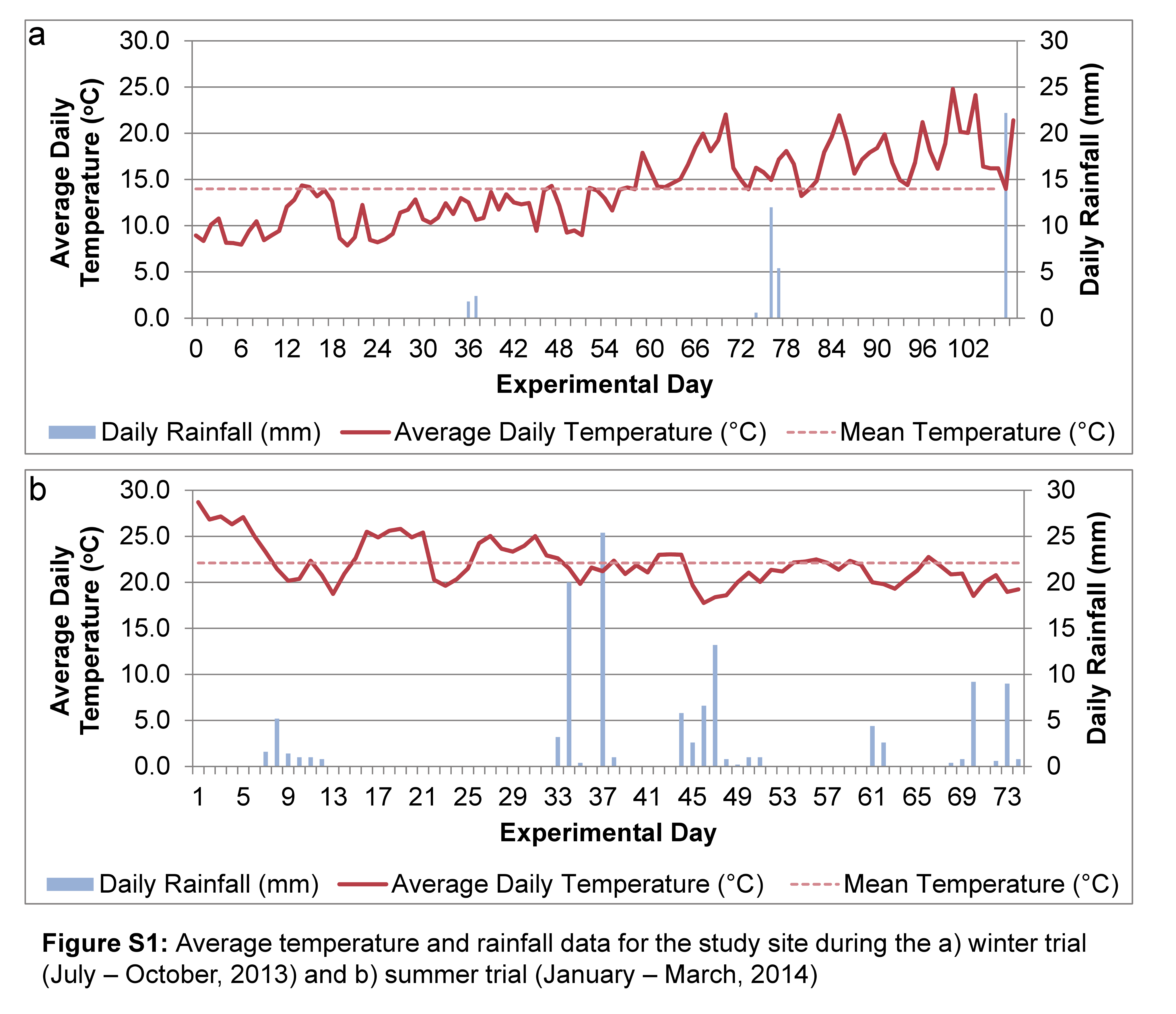

Supplement: Figure S1 — Average temperature and rainfall data for the study site during the a) winter trial (July – October, 2013) and b) summer trial (January – March, 2014). (TIF) [file pone.0113681.s001.tif]
