# Supplementary material for: Comparison of the Decomposition VOC Profile during Winter and Summer in a Moist, Mid-Latitude (Cfb) Climate
Source: PLoS One. 2014 Nov 20;9(11):e113681. doi: 10.1371/journal.pone.0113681 (PMC4239107; doi:10.1371/journal.pone.0113681)
Supplement: Appendix S1 — Decomposition VOCs detected during winter (O) and summer (X) for each stage of decomposition. (DOCX) [file pone.0113681.s002.docx]

**Appendix 1: Decomposition VOCs detected during winter (O) and summer (X) for each stage of decomposition**

|  | **FRESH*** | **BLOAT** | **ACTIVE DECAY** | **ADVANCED DECAY** | **DRY/REMAINS** |
| --- | --- | --- | --- | --- | --- |
| ***Sulfur-containing compounds*** |  |  |  |  |  |
| 1-Propanol, 3-(methylthio)- |  |  | X |  |  |
| 2,4-Dithiapentane |  |  | X | X | X |
| Aminomethanesulfonic acid |  |  | X |  | X |
| Cyclohexane, isothiocyanato- |  |  |  | XO |  |
| Dimethyl disulfide |  | XO | XO | XO | X |
| Dimethyl pentasulfide |  |  | XO |  |  |
| Dimethyl sulfide |  |  |  | XO |  |
| Dimethyl sulfone |  |  | X |  |  |
| Dimethyl sulfoxide |  |  | X |  |  |
| Dimethyl tetrasulfide |  |  | X | X |  |
| Dimethyl trisulfide |  | XO | XO | XO | X |
| Disulfide, methyl (methylthio) methyl |  |  | X | X |  |
| Disulfide, methyl propyl |  |  | X | X |  |
| Formamide, N-methylthio |  |  | X | X |  |
| Heptane, 1-(methylthio)- |  |  | X | X | X |
| Hexane, 1-(methylthio)- |  |  | X | X |  |
| Mercaptoacetone |  |  | X | X |  |
| Methane, isothiocyanato- |  |  | XO | X |  |
| Methanethiol |  |  | XO | X | X |
| Methyl ethyl disulfide |  |  | X | X | X |
| Methyl isopropyl disulphide |  |  |  |  | X |
| Methyl propanethioate |  |  | X |  |  |
| Methyl thiolacetate |  |  |  | X |  |
| Pentane, 1-(methylthio)- |  |  | X |  | X |
| Sulfide, isobutyl isopropyl |  |  | X |  |  |
| Sulfur dioxide |  |  | X | X | X |
| Thiophene, 2-pentyl- |  |  | X | X |  |
|  | **FRESH** | **BLOAT** | **ACTIVE DECAY** | **ADVANCED DECAY** | **DRY/REMAINS** |
| ***Nitrogen-containing compounds*** |  |  |  |  |  |
| 1,2-Benzenediamine, N-methyl- |  |  |  |  | X |
| 1,3,5-Triazine |  |  | XO |  |  |
| 1-Butanamine, 3-methyl- |  |  | X |  |  |
| 1-Butanamine, 3-methyl-N-(3-methylbutylidene)- |  |  | X | X | X |
| 1H-Indole, 2-methyl- |  |  | X |  |  |
| 1H-Pyrrole, 1-methyl- |  |  | X |  |  |
| 1H-Pyrrole, 2,3-dimethyl- |  |  | X |  |  |
| 1H-Pyrrole, 2,5-dimethyl- |  |  |  | X |  |
| 1-Nonadecanamine, N,N-dimethyl- |  |  |  |  | X |
| 1-Propanamine, N,2-dimethyl-N-nitroso- |  |  | X | X |  |
| 2-(Diethylamino)acetonitrile |  |  |  |  | X |
| 2-Ethynyl pyridine |  |  |  | XO |  |
| 2-Piperidinone |  |  | X |  | X |
| 2-Propyn-1-amine, N,N-dimethyl- |  |  | X |  |  |
| 5H-1-Pyridine |  |  | XO | XO | X |
| Acetamide, N-4-pyridinyl- |  |  |  |  | X |
| Acetonitrile, (dimethylamino)- |  |  | X | X | X |
| Acetonitrile, hydroxy- |  |  |  | X |  |
| Aziridine, 2-isopropyl-1,3-dimethyl-, trans- |  |  |  | X |  |
| Benzamide, N,N-diethyl-4-methyl- |  | XO |  | XO |  |
| Benzonitrile |  |  | XO | XO | X |
| Butanamide |  |  | X |  |  |
| Butanamide, 3-methyl- |  |  |  | X | X |
| Butanenitrile, 3-methyl- |  |  |  | X |  |
| Dimethylamine |  |  | XO |  |  |
| Ethenamine, N-methylene- |  |  | X |  |  |
| Formamide, N,N-dimethyl- |  |  |  | X |  |
| Formamide, N-butyl- |  |  |  | X | X |
|  | **FRESH** | **BLOAT** | **ACTIVE DECAY** | **ADVANCED DECAY** | **DRY/REMAINS** |
| Formamide, N-methyl- |  |  | X |  |  |
| Hexanenitrile |  |  |  |  | X |
| Indole |  |  | X | X |  |
| Isoxazole, 5-methyl- |  |  | X |  |  |
| Methenamine |  | O | XO | XO | X |
| Methyl dimethylcarbamate |  |  | X | X |  |
| Methylamine, N,N-dimethyl- |  | O | XO | XO | X |
| N,N-Dimethylacetamide |  |  | X |  |  |
| Pyrazine, 2,5-dimethyl- |  |  |  | X |  |
| Pyrazine, 2,6-dimethyl- |  |  | X | X |  |
| Pyrazine, methyl- |  |  | X |  |  |
| Pyrazine, trimethyl- |  |  | X | XO |  |
| Pyridine |  |  |  |  | X |
| Pyridine, 2-methyl- |  |  | X |  | X |
| Pyridine, 3-methyl- |  |  | X |  | X |
| Pyrrole |  |  | X | X |  |
| ***Aromatics*** |  |  |  |  |  |
| 2-n-Butyl furan |  |  |  |  | X |
| Benzene |  | O | X | XO | X |
| Benzene, 1-methyl-3-(1-methylethyl)- |  |  |  | X |  |
| Ethylbenzene |  | O | X | O |  |
| Furan |  |  |  | XO |  |
| Furan, 2,3-dihydro- |  |  |  |  | X |
| Furan, 2-hexyl- |  |  |  |  | X |
| Furan, 2-pentyl- |  |  | XO | XO | XO |
| o-Xylene |  |  |  | XO |  |
| Phenol |  |  | XO | XO | X |
| Styrene |  |  | X |  |  |
| Toluene |  |  | X | XO |  |
|  | **FRESH** | **BLOAT** | **ACTIVE DECAY** | **ADVANCED DECAY** | **DRY/REMAINS** |
| ***Esters*** |  |  |  |  |  |
| 2-Propenoic acid, methyl ester |  |  |  | X | X |
| Acetic acid, anhydride with formic acid |  |  | X |  |  |
| Acetic acid, butyl ester |  |  | XO |  |  |
| Acetic acid, hydroxy-, ethyl ester |  |  |  | XO |  |
| Acetic acid, methyl ester |  |  | X | XO | X |
| Acetic acid, oxo-, methyl ester |  |  | X | O |  |
| Benzoic acid, methyl ester |  | O | XO | XO |  |
| Butanoic acid, 1-methylethyl ester |  |  | X | X |  |
| Butanoic acid, 1-methylpropyl ester |  |  | X |  |  |
| Butanoic acid, 2-methyl-, ethyl ester |  |  | X | X |  |
| Butanoic acid, 2-methyl-, methyl ester |  |  | X |  |  |
| Butanoic acid, 3-methyl-, ethyl ester |  |  | X |  |  |
| Butanoic acid, butyl ester |  |  | X | O |  |
| Butanoic acid, ethyl ester |  |  | X | X |  |
| Butanoic acid, methyl ester |  |  | XO | XO |  |
| Butanoic acid, propyl ester |  |  | X |  |  |
| Carbonic acid, butyl phenyl ester |  |  |  |  | X |
| Carbonic acid, dimethyl ester |  |  | XO | X |  |
| Decanoic acid, methyl ester |  |  |  | XO |  |
| Ethyl Acetate |  |  |  | XO |  |
| Formic acid, heptyl ester |  |  |  |  | X |
| Hexadecanoic acid, methyl ester |  |  |  |  | X |
| Isopropyl acetate |  |  | X |  |  |
| Isopropyl myristate |  |  |  | XO |  |
| Methyl isovalerate |  |  | X | X | X |
| Methyl propionate |  |  | X | XO |  |
| Methyl thiolacetate |  |  | XO |  |  |
| n-Propyl acetate |  |  | X | X |  |
|  | **FRESH** | **BLOAT** | **ACTIVE DECAY** | **ADVANCED DECAY** | **DRY/REMAINS** |
| Propanoic acid, 1-methylethyl ester |  |  | X |  |  |
| Propanoic acid, 2-methyl-, ethyl ester |  |  | X |  |  |
| Propanoic acid, ethyl ester |  |  | X |  |  |
| Propanoic acid, propyl ester |  |  | X |  |  |
| sec-Butyl acetate |  |  | X |  |  |
| Sulfurous acid, dimethyl ester |  |  |  | X |  |
| Thiocyanic acid, methyl ester |  |  | XO | X | X |
| ***Alcohols*** |  |  |  |  |  |
| 3-Methyl-2-butanol |  |  |  | X | X |
| 2-Pentanol |  |  | X | XO |  |
| 1,5-Hexadien-3-ol |  |  |  | X |  |
| 1-Butanol |  |  | XO | XO | O |
| 1-Butanol, 3-methyl- |  |  | XO | XO | X |
| 1-Butanol, 3-methyl-, formate |  |  |  |  | X |
| 1-Heptanol |  |  | X |  |  |
| 1-Hepten-3-ol |  |  |  | X | X |
| 1-Hexadecanol |  |  |  | XO |  |
| 1-Hexanol |  |  | X | X | X |
| 1-Hexanol, 2-ethyl- |  |  |  |  | X |
| 1-Octanol |  |  |  |  | X |
| 1-Octen-3-ol |  |  | X | XO | X |
| 1-Pentanol |  |  | X | X | X |
| 1-Penten-3-ol |  |  | X | X |  |
| 1-Propanol |  | XO | XO | XO | O |
| 1-Propanol, 2-methyl- |  |  | XO | XO | X |
| 2-Butanol |  |  | XO | XO |  |
| 2-Butanol, 3-methyl- |  |  | X |  |  |
| 2-Buten-1-ol, 3-methyl- |  |  | X |  |  |
| 2-Heptanol, (S)- |  |  |  | X |  |
|  | **FRESH** | **BLOAT** | **ACTIVE DECAY** | **ADVANCED DECAY** | **DRY/REMAINS** |
| 2-Heptanol, 2-methyl- |  |  | X |  |  |
| 2-Hexanol |  |  | X | X |  |
| 2-Pentanol |  |  |  | X |  |
| 2-Pentanol, acetate |  |  | X |  |  |
| 3,5-Dithiahexanol 5,5-dioxide |  |  |  | XO |  |
| 3-Buten-1-ol, 3-methyl- |  |  | X |  |  |
| 3-Butyn-2-ol |  |  |  | X |  |
| 3-Hexen-1-ol, acetate |  |  |  | XO | XO |
| 3-Pentanol |  |  |  | X | X |
| Benzyl alcohol |  |  |  |  | X |
| Cyclobutane methanol |  |  |  | X |  |
| Ethanol | O | O | XO | XO |  |
| Ethanol, 2-ethoxy- |  |  | XO |  |  |
| Ethanol, 2-phenoxy- |  |  |  | XO |  |
| Eucalyptol |  |  | X |  |  |
| Isopropyl alcohol |  | XO | XO | XO |  |
| p-Cresol |  |  | X | X |  |
| Phenylethyl alcohol |  |  |  |  | X |
| ***Ketones*** |  |  |  |  |  |
| 1,4-Pentadien-3-one |  |  | XO |  |  |
| 1-Octen-3-one |  |  | X | XO | X |
| 1-Penten-3-one |  |  | X | X |  |
| 2,3-Butanedione |  |  | X | XO | X |
| 2,3-Hexanedione |  |  |  | X | X |
| 2,3-Octanedione |  |  |  | X | X |
| 2,3-Pentanedione |  |  | X | X | X |
| 2-Butanone | O | O | XO | XO | X |
| 2-Butanone, 3-methyl- |  |  | XO | XO |  |
| 2-Decanone |  |  |  | X | X |
|  | **FRESH** | **BLOAT** | **ACTIVE DECAY** | **ADVANCED DECAY** | **DRY/REMAINS** |
| 2-Heptanone |  |  | X | XO | X |
| 2-Heptanone, 6-methyl- |  |  |  |  | X |
| 2-Hexanone |  |  | X | X | X |
| 2-Hexanone, 5-methyl- |  |  |  | X |  |
| 2-Nonanone |  |  |  | XO | X |
| 2-Octanone |  |  |  | XO | X |
| 2-Oxetanone, 4-methyl- |  |  | XO |  |  |
| 2-Pentanone | O | O | XO | XO | X |
| 2-Pentanone, 3-methyl- |  |  | X | X |  |
| 2-Pentanone, 4-hydroxy-4-methyl- |  |  |  | XO |  |
| 2-Tetradecanone |  |  |  |  | X |
| 2-Undecanone |  |  |  |  | X |
| 3-Heptanone |  |  |  |  | X |
| 3-Hexanone |  |  | X | X |  |
| 3-Octanone |  |  | X | X | X |
| 3-Octen-2-one |  |  |  |  | X |
| 3-Pentanone |  | O | O | XO |  |
| 3-Pentanone, 2,4-dimethyl- |  |  |  |  | X |
| 3-Penten-2-one |  |  | X | X |  |
| 3-Penten-2-one, 4-methyl- |  |  |  | XO |  |
| 5-Hepten-2-one, 6-methyl- |  |  |  |  | X |
| Acetophenone |  |  | XO | XO | X |
| Cyclobutanone, 3-ethyl- |  |  | X |  |  |
| Cyclopentanone |  |  |  |  | X |
| Ethanone, 1-cyclopropyl- |  |  |  |  | X |
| Methyl isobutyl ketone |  |  | XO | XO | X |
| Methyl vinyl ketone |  | O | XO | XO | X |
| ***Aldehydes*** |  |  |  |  |  |
| 2-Butenal, 2-ethyl- |  |  | X | X | X |
|  | **FRESH** | **BLOAT** | **ACTIVE DECAY** | **ADVANCED DECAY** | **DRY/REMAINS** |
| 2-Butenal, 2-methyl- |  |  | X |  |  |
| 2-Butenal, 3-methyl- |  |  | X | X | X |
| 2-Decenal |  |  |  |  | X |
| 2-Ethylacrolein |  |  | XO |  |  |
| 2-Heptenal |  |  | X | X | X |
| 2-n-Butylacrolein |  |  | O |  | X |
| 2-Nonenal |  |  |  |  | X |
| 2-Octenal |  |  |  |  | X |
| 2-Propenal |  |  |  | XO |  |
| Benzaldehyde |  | O | XO | XO | X |
| Butanal |  |  | XO | XO |  |
| Butanal, 2-methyl- |  |  | X | X | X |
| Butanal, 3-methyl- |  |  | XO | XO | X |
| Decanal |  |  | XO | XO |  |
| Formaldehyde |  |  | X | X | X |
| Heptanal |  |  | X | XO | XO |
| Hexanal |  |  | X | XO | XO |
| Methacrolein |  |  | X |  | X |
| Methylal |  |  |  |  | X |
| Nonanal |  | O |  | XO | XO |
| Octanal |  |  |  | O | XO |
| Pentanal |  |  | X | XO | X |
| Pentanal, 2-methyl- |  |  |  | X |  |
| Propanal |  |  |  | X |  |
| Propanal, 2-methyl- |  |  | X | X | X |
| ***Carboxylic acids*** |  |  |  |  |  |
| Acetic acid |  |  |  | X | X |
| Butanoic acid |  |  | X | XO | XO |
| Butanoic acid, 2-methyl- |  |  |  | XO | X |
|  | **FRESH** | **BLOAT** | **ACTIVE DECAY** | **ADVANCED DECAY** | **DRY/REMAINS** |
| Butanoic acid, 3-methyl- |  |  | X | XO | X |
| Hexanoic acid |  |  |  |  | X |
| Hexanoic acid, 2-methyl- |  |  |  | X |  |
| Pentanoic acid |  |  |  | X | X |
| Propanoic acid |  |  | X |  | X |
| Propanoic acid, 2-methyl- |  |  | X | XO | X |
| ***Hydrocarbons*** |  |  |  |  |  |
| 1,3,7-Octatriene, 3,7-dimethyl- |  |  |  |  | X |
| 1,3-Dioxolane, 2-methyl- |  |  | O | XO | X |
| 1,3-Octadiene |  |  |  |  | X |
| 1,4-Cyclohexadiene, 1-methyl- |  |  |  |  |  |
| 1,4-Dioxane |  |  | XO | XO | X |
| 1,4-Dioxin, 2,3-dihydro- |  |  |  | X |  |
| 1-Heptene |  |  |  |  | X |
| 1-Octene |  |  |  | X | X |
| 2,2-Dimethoxybutane |  |  | X | X |  |
| 2,2-Dimethyl-1-aza-spiro[2.3]hexane |  |  | X | X |  |
| 2-Heptene, 3-methyl- |  |  |  |  | X |
| 2-Octene |  |  | X |  | X |
| 3-Heptene, 3-methyl- |  |  |  | O | X |
| 3-Octene |  |  |  |  | X |
| 8-Heptadecene |  |  |  | O | X |
| à-Phellandrene |  |  | X | X | X |
| á-Pinene | O | O | XO | XO | O |
| Bicyclo[3.1.0]hex-2-ene, 4-methyl-1-(1-methylethyl)- |  |  |  | X |  |
| Bicyclo[4.2.0]octa-1,3,5-triene |  |  |  | XO |  |
| Camphene |  |  | O | XO | O |
| cis-2-Methyl-7-octadecene |  |  |  | X | X |
| Cyclohexane, ethyl- |  |  |  |  | X |
|  | **FRESH** | **BLOAT** | **ACTIVE DECAY** | **ADVANCED DECAY** | **DRY/REMAINS** |
| Cyclohexane, propyl- |  |  |  |  | X |
| Cyclohexene, 1-methyl-4-(1-methylethenyl) |  |  |  | X |  |
| Cyclohexene, 3,3,5-trimethyl- |  |  |  |  | X |
| Cyclopentane, 1-ethyl-2-methyl- |  |  |  | XO |  |
| Cyclopropane, ethyl- |  |  |  | O | X |
| Cyclopropane, ethylidene- |  |  |  | X |  |
| Decane, 3-methyl- |  |  |  | XO |  |
| Dodecane |  |  |  |  | X |
| Eicosane | O |  |  | X | XO |
| Ethane |  | XO |  |  |  |
| Heptadecane |  |  |  | X |  |
| Heptane |  |  | X | XO | XO |
| Heptane, 3-ethyl-2-methyl- |  |  |  |  | X |
| Hexadecane | O | O | XO | XO |  |
| Hexane, 2-methyl-4-methylene- |  |  |  | XO |  |
| Limonene |  |  | XO | X | O |
| n-Hexane |  | O | X | XO | X |
| Nonadecane |  |  |  | O | X |
| Nonane |  |  |  |  | X |
| Nonane, 2,2,4,4,6,8,8-heptamethyl- |  |  |  |  | X |
| Octane |  |  | X | XO | XO |
| Octane, 2,4,6-trimethyl- |  |  |  |  | X |
| o-Cymene |  |  | O | XO | O |
| Pentadecane |  | O |  | X | XO |
| Pentane |  |  |  | XO | X |
| Pentane, 3-methyl- |  |  |  |  | X |
| Tetradecane |  |  |  | O | X |
| trans-calamenene |  |  |  | XO |  |
| Tridecane |  |  |  |  | X |
|  | **FRESH** | **BLOAT** | **ACTIVE DECAY** | **ADVANCED DECAY** | **DRY/REMAINS** |
| Undecane | O | O |  | XO | X |
| ***Ethers*** |  |  |  |  |  |
| 2,2-Dimethoxybutane |  | XO | XO | XO |  |
| 3-Methoxybut-1-ene |  |  |  | XO |  |
| Anisole |  |  |  |  | X |
| Benzene, 1,3,5-trimethoxy- |  |  |  |  | X |
| Butane, 1-methoxy- |  |  | XO |  |  |
| Butane, 1-methoxy-3-methyl- |  |  | O |  | X |
| Hexane, 1-methoxy- |  |  |  |  | X |
| Propane, 1,1-dimethoxy- |  |  |  | XO |  |
| Propane, 1,1-dimethoxy-2-methyl- |  |  |  | XO |  |
| Propane, 2,2-dimethoxy- |  |  | XO | XO |  |

*VOC samples were not collected during the fresh stage of the summer trial due to rapid decompostion
